# Automated, high-throughput measurement of size and growth curves of small organisms in well plates

**DOI:** 10.1038/s41598-018-36877-0

**Published:** 2019-01-09

**Authors:** James Duckworth, Tjalling Jager, Roman Ashauer

**Affiliations:** 1 Environment Department, University of York, Wentworth Way, Heslington, York, YO10 5NG United Kingdom; 2 DEBtox research, De Bilt, The Netherlands

## Abstract

Organism size and growth curves are important biological characteristics. Current methods to measure organism size, and in particular growth curves, are often resource intensive because they involve many manual steps. Here we demonstrate a method for automated, high-throughput measurements of size and growth in individual aquatic invertebrates kept in microtiter well-plates. We use a spheroid counter (Cell^3^iMager, cc-5000) to automatically measure size of seven different freshwater invertebrate species. Further, we generated calibration curves (linear regressions, all p < 0.0001, r^2^ >=0.9 for *Ceriodaphnoa dubia*, *Asellus aquaticus*, *Daphnia magna*, *Daphnia pulex;* r^2^ >=0.8 for *Hyalella azteca*, *Chironomus spec*. larvae and *Culex spec*. larvae) to convert size measured on the spheroid counter to traditional, microscope based, length measurements, which follow the longest orientation of the body. Finally, we demonstrate semi-automated measurement of growth curves of individual daphnids (*C*. *dubia* and *D*. *magna*) over time and find that the quality of individual growth curves varies, partly due to methodological reasons. Nevertheless, this novel method could be adopted to other species and represents a step change in experimental throughput for measuring organisms’ shape, size and growth curves. It is also a significant qualitative improvement by enabling high-throughput assessment of inter-individual variation of growth.

## Introduction

Organism size and growth curves are important biological characteristics. They are frequently measured in a wide range of species and disciplines, for example in ecology, physiology and ecotoxicology. Measurements of organism size and growth over time inform theory development, such as the Metabolic Theory of Ecology^1,2^ or Dynamic Energy Budget Theory^3,4^, and more efficient methods to generate such empirical data would significantly increase our ability to test those theories in a wider range of species and develop them further. Measuring organism growth over time is also important to understand the toxicity of man-made chemicals^5,6^, but, in regulatory chemical safety testing, size is only measured at the end of the test^7^. Slower growth and reduced organism size due to chemical exposure can lead to ecological impacts, for example by delaying maturity and reducing reproductive output (since body size is linked to feeding rates) or related size effects, as prey or predator. Due to the large number of chemical structures known (>140 million, https://support.cas.org/) and more than 100’000 on the market^8^ (https://echa.europa.eu/information-on-chemicals/ec-inventory), in combination with the huge number of species in the environment, it is important to speed up toxicity testing, if possible by automation. This trend towards high-throughput testing is manifest mostly in the many *in-vitro* toxicity tests^9,10^. However, high-throughput testing of chemical effects on growth, for example in a range of aquatic invertebrate species, would generate much needed information on potential effects of pollutants on organism physiology, which is difficult to assess based on *in-vitro* tests^11,12^. Toxicants interfere with energy fluxes in organisms in distinct ways, so called physiological modes of action^5,12^, and to identify these we need observations on body size and reproduction over a good part of the life cycle^6^. We currently lack a systematic understanding of the physiological modes of toxic action across chemicals and species^12^. This lack of knowledge is partly due to the resources required to measure growth curves.

Recently a range of new methods has been developed to partially automate or otherwise improve measurements of organism size^11,13–19^. Yet some important limitations remain. Methods based on microscopy, photography or scanning, with subsequent software based size measurements, can still be relatively time consuming and labor intensive, which is a limiting factor for research questions that aim at finding patterns across large numbers of chemicals and species. Laser optical plankton counting^18^ does not permit repeated measurements on the same individuals without removing them from their growth vessel. The frequent use of custom build equipment is another hurdle towards widespread adoption. Furthermore, transfer of organisms into a specific vessel for measurement, e.g. onto microscope slides, can be stressful and disruptive for test organisms. Bulk image capture of whole cohorts prohibits repeated measurements on the same individual. And, to the best of our knowledge, methods for repeatedly and automatically measuring the size of the same individual organism, specifically for aquatic invertebrates, are still missing.

Hence the aim of this study is to develop a method for automated, non-destructive, high-throughput measurements of size and growth in individual aquatic invertebrates kept in microtiter well-plates. Specifically we (i) use a commercially available machine, the spheroid counter Cell^3^iMager (cc-5000, SCREEN Holdings Co. Ltd., Kyoto, Japan), to measure size related parameters in a range of aquatic invertebrate species and calibrate those measurements against traditional size measurements using a microscope, and (ii) demonstrate its suitability for measuring growth curves of individuals over time.

## Results

### Calibration of size measurements

Comparisons between organism diameter determined with the spheroid counter and microscope measured length, revealed that in four of the seven tested species (*Ceriodaphnoa dubia*, *Asellus aquaticus*, *Daphnia magna*, *Daphnia pulex*) the correlation between the two values was high (r^2^ >=0.9), while for the three other species (*Hyalella azteca*, *Chironomus spec*. larvae and *Culex spec*. larvae) the correlation was weaker (r^2^ < 0.8, Table 1). The slopes of the linear relationships between the diameter measured with the spheroid counter and the organism length measured manually under a microscope were all significantly different from zero (p < 0.0001). The length measurements under the microscope accounted for the species specific body shape and correlations decrease from species with roughly spherical body shapes (*C*. *dubia*, *A*. *aquaticus*, *D*. *magna*, *D*. *pulex*) to species with elongated bodies (*H*. *azteca*, *Chironomus sp*. larvae, *Culex spec*. larvae). Thus estimating body length from spheroid counter measurements will be more precise for spherical species. It should be noted however, that for many biological questions (e.g. dynamic energy budget modelling^20,21^), body length is not the most relevant endpoint; it is used as a proxy for body mass, which has a direct link to the bioenergetics^20^. We focus here on comparing the spheroid counter results to length measurement by microscopy, but for application in a bioenergetics context, some of the other variables measured by the spheroid counter, or combinations of variables, may be more relevant (especially if they can be used to estimate body volume, which is a better proxy for body mass than total length).

**Table 1:** Calibration of automated size measurement.

Species	Regression equation	r^2^	n
*Ceriodaphnia dubia*	y = 1.29 × − 14.7	0.930	211
*Asellus aquaticus*	y = 1.50 × + 86.0	0.928	70
*Daphnia magna*	y = 1.56 × − 57.8	0.912	449
*Daphnia pulex*	y = 1.26 × + 257	0.900	271
*Hyalella azteca*	y = 2.43 × + 415	0.785	174
*Chironomus sp*. larvae	y = 2.95 × + 1790	0.707	80
*Culex sp*. larvae	y = 1.08 × + 1370	0.422	122

Linear relationships between spheroid counter diameter (x, µm) and microscope measured length (y, µm) for the study species (all p < 0.0001).

**Figure 1:**
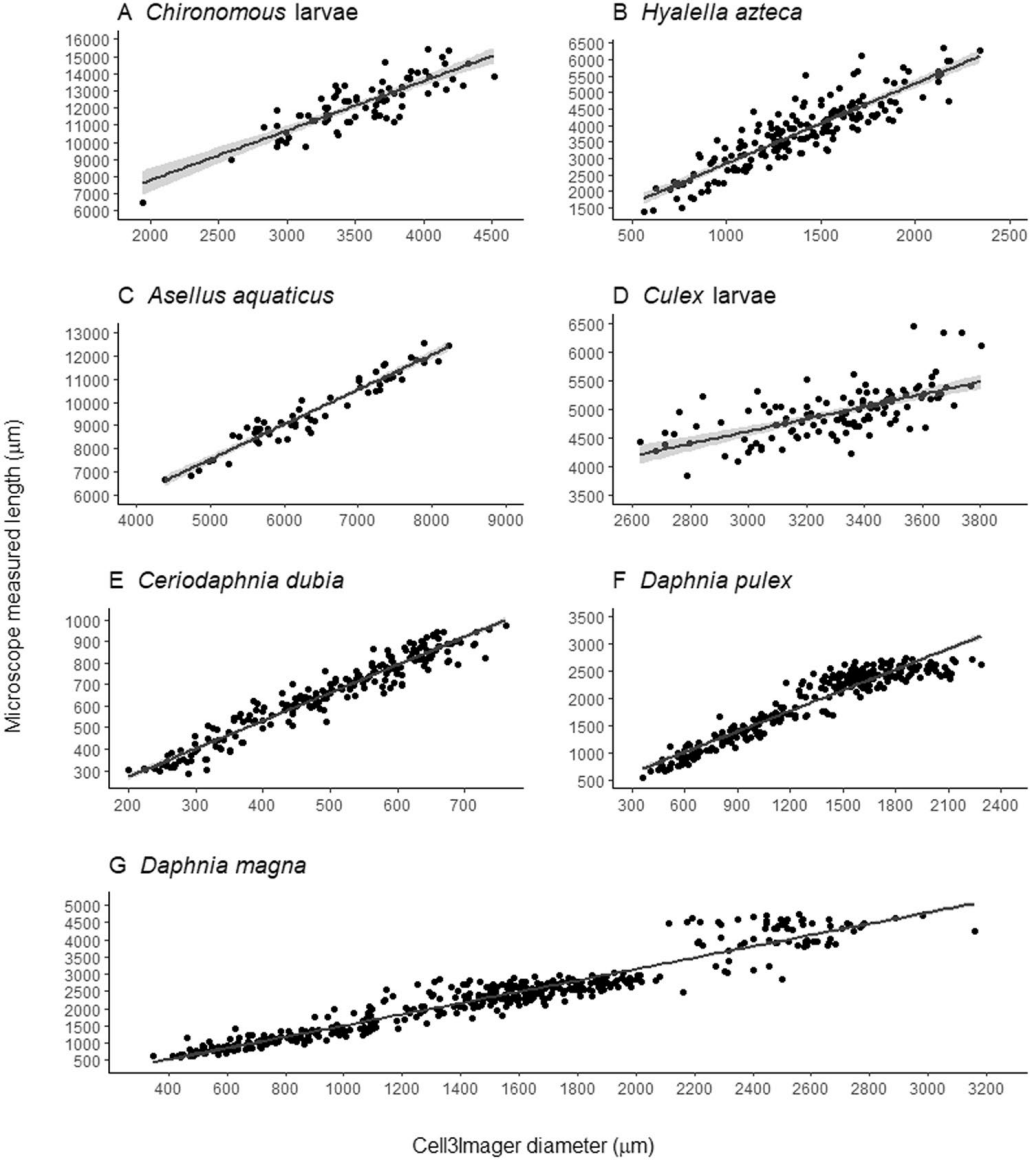
Spheroid counter (Cell3imager) measured diameter against microscope measured length. Each panel shows a different species with a regression line representing the linear relationship between the two measured variables (the shaded grey areas show the 95% Confidence interval for the line of best fit). Regression line equations can be found in Table 1.

The calibration curves for *C*. *dubia*, *A*. *aquaticus*, *D*. *magna*, *D*. *pulex*, *H*. *azteca*, *Chironumus spec*. larvae and *Culex spec*. larvae show the linear relationships between the spheroid counter measured diameter and microscope measured length (Fig. 1). Regression equations can be used to convert diameter measurements from the spheroid counter into traditional length measurements obtained via microscopy. Values for the spheroid counter are always smaller than those produced via microscopy because the spheroid counter measures diameter as an average across the shape, by converting the shape into a circle with the same area, and because organisms are randomly oriented in the well plate medium, not necessarily exposing their profile to the scanner. Conversely, traditional microscopy measures length along the longest orientation of the organism.

### Growth curves

**Figure 2:**
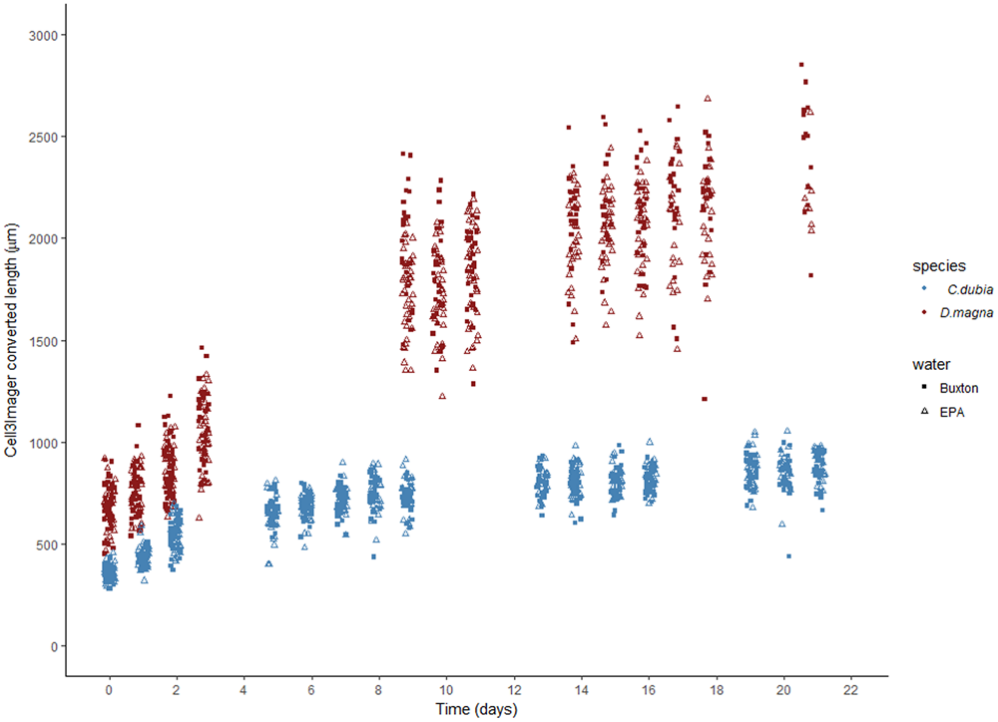
Automated measurement of growth curves demonstrated using two cohorts of daphnid species (*D*. *magna* and *C*. *dubia*). Spheroid counter (Cell^3^iMager) converted length shows the predicted length as if it had been measured using a microscope. Each individual point is plotted over time and jittered to avoid overcrowding of points (red: *D*. *magna*, blue: *C*. *dubia*). Two different media were tested (solid squares: Buxton spring water, open triangles: EPA standard freshwater).

Growth experiments were carried out over a period of 21 days to create a growth curve for both *C*. *dubia* and *D*. *magna*. *C*. *dubia* showed the most growth during the first five days in treatment, then size remained approximately constant for the remainder of the experiment. *D*. *magna* grew fast in the initial four days, before not being recorded for five days (long weekend for experimenter), and then continued to steadily increase in size to the end of the experiment (Fig. 2). Figure 2 shows repeated size measurements on the same cohort of individuals. Size was converted from spheroid counter measured diameter to organism length using the species specific regressions from Table 1 (i.e. converted length = y).

Occasionally, data for certain wells could not be gathered due to organisms hiding in the edges of the well plates or moving too fast to be scanned. Therefore, the sample size on each day varied slightly. Mortality was very low across each plate (<10%), except for day 21 in *D*. *magna*. Thus fewer data points were available for this observation time point and data for day 22 is omitted due to too few organisms being alive. This was likely due to the induction of stress through the infection of the well plates by a microorganism, which turned the water in most of the wells green.

## Discussion

We have established methods to automatically measure size of seven different freshwater invertebrate species and established calibration curves (linear regressions) to convert size measured on the spheroid counter to traditional length measurements which follow the longest orientation of the body. Once such a regression is established, it can be used to convert future size measurements in different experiments with that species. The precision depends on the species’ body shape and decreases as organism morphology deviates from spherical to elongated body shapes. If accurate characterization of the body shape and 3D geometry is important, then other methods, such as those based on confocal microscopy^19^, would be more appropriate.

**Figure 3:**
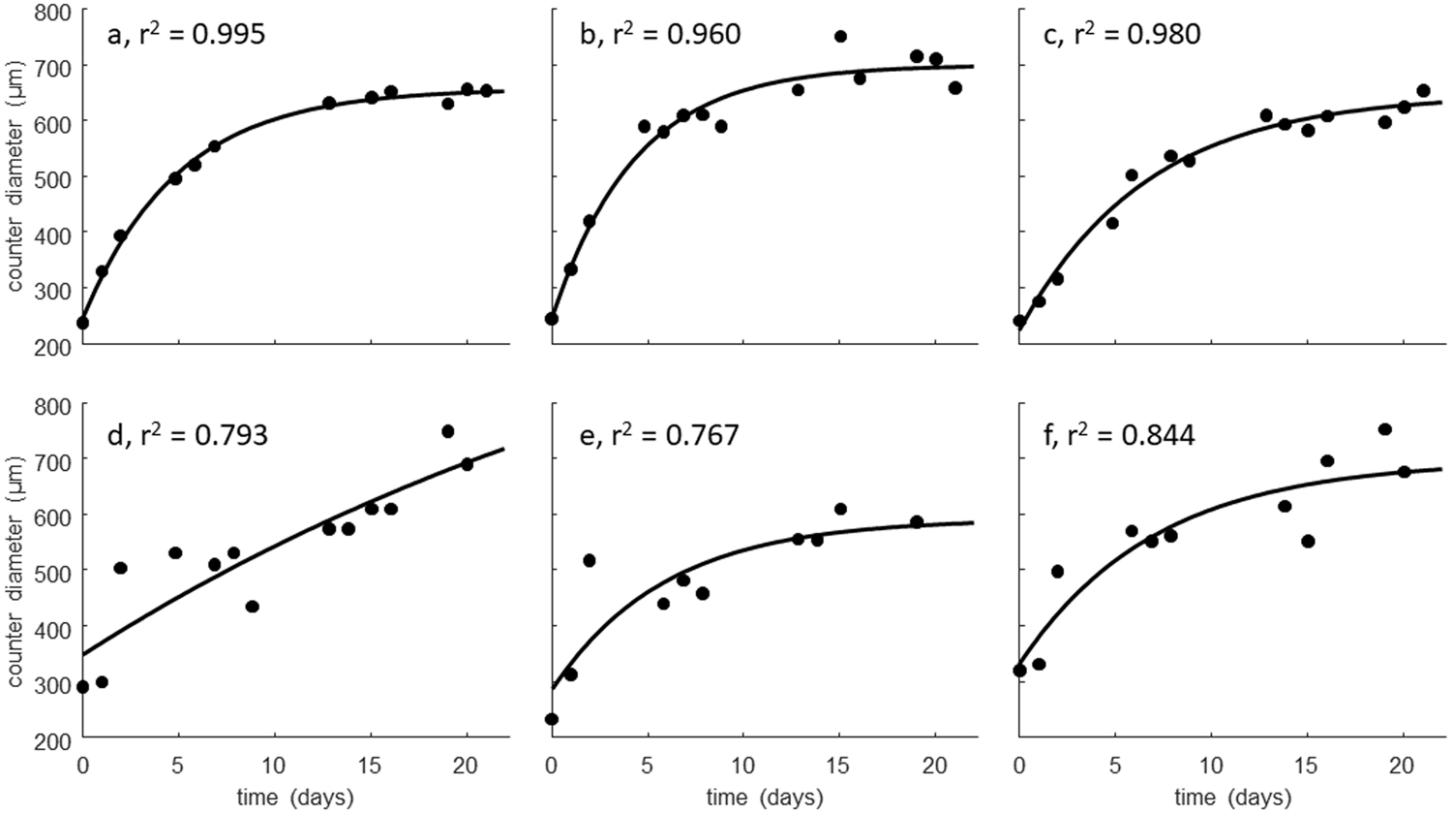
Examples of von Bertalanffy growth curves [*L*(*t*) = *L*_∞_ − (*L*_∞_ − *L*_0_) exp (−*rt*)] fitted to body size measured over time for individual daphnids (*C*. *dubia*). Top figures show excellent (**a**) to good (**b**,**c**) fits, whereas the bottom row shows some very poor fits (**d**–**f**).

The growth curves demonstrate that the spheroid counter can be used to repeatedly measure size and therefore growth of the same individuals over time, which is important for understanding inter-individual variation in life-history responses to stress^22^. Using average size of the individuals over time can bias growth estimates^22^, hence we here demonstrate that growth curves for individual daphnids can be derived from spheroid counter data (Fig. 3).

The automatically measured size of the freshwater organisms can be converted to body length by application of species-specific regression equations. However, in Fig. 3, we used the organism diameter as measured by the spheroid counter, which may be a better proxy for body mass than total organism length. Most of the time series for diameter of individuals were well-described by the von Bertalanffy curve (Fig. 3, top row), although the occurrence of unrealistic time series (Fig. 3, bottom row) shows that some fine tuning of the method is needed.

**Figure 4:**
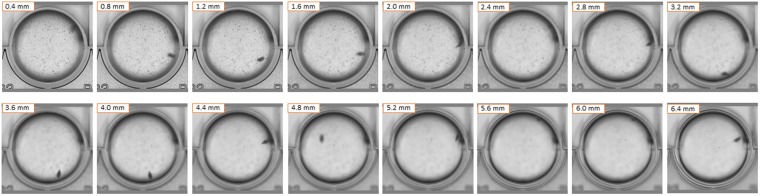
Images of a bracket scan of well A3 at 234 h of the growth curve experiment. There are 16 images taken shortly after each other (ca. 100 seconds) and at different focus heights (0.4 mm increments). Note the different position and orientation of the same *D*. *magna* individual in the same well.

This experiment shows, that with current methodology, long term exposure experiments of at least 20 days could be carried out with daphnids and that individual growth curves can be estimated. However the quality of the growth curve fit varies strongly amongst individuals in our experiment (See examples in Fig. 3). Clearly, not all of this variability is natural. We suspect that it is largely due to new sources of variability introduced by this method, specifically the free three-dimensional orientation and movement of organisms in the wells combined with semi-automated image analysis. The bracket scans slice the well into several layers in the z-axis (see Fig. 4), but between each image capture some time passes (several tens of seconds to a few minutes, depending on e.g. settings, number of wells scanned, scan resolution). Thus the software or the operator (both are options) then have to select the best focus image from a series of images of the same well, taken shortly after each other (ca. 100 seconds in our growth experiment), with the organism being more in focus in some images than others, but crucially also with different orientation and positioning of the organism in relation to the image plane and focus height. These sources of error should be reduced, but this requires further research.

Further research is needed to refine the method presented here, as it still has some limitations. The selection of the image that is most in focus per each well was still done manually in this study, but could be automated in the future. Occasionally the spheroid counter failed to capture an image of an individual, for example when they moved in a specific vertical pattern and so by chance avoided all bracket scans of a given well. However, starting the experiments with large sample sizes can minimize the impact of this for average population growth curves but not individual growth curves. In addition, there are also new opportunities offered by the types of measurements possible with the spheroid counter, such as measuring circularity to detect growth deformities or studying offspring size in multigenerational tests. There are also some advantages in addition to high-throughput. Using the spheroid counter to measure freshwater organisms is the least invasive way to measure organisms, as there is no point where the individual has be removed from water. It also reduces potential sources of variability and human error by automating measurements, and images are stored for further analysis.

The daphnids in our growth curve experiments were females, freshly hatched from ephippia rather than being sourced from highly standardized parthogenetic cultures as is common practice in regulatory ecotoxicity testing^23^. Nutrient ratios in the daphnia food pellets used here also differ from those in other food types (see supporting information), thus constituting another source of variability in daphnid growth^24–26^. Thus the growth curves in Fig. 2 can be expected to differ from those measured elsewhere under different conditions. What we demonstrated here is a new and faster method to generate growth curves for daphnids, and possibly other species amenable to growing in well plates. This step change in experimental throughput will enable larger multifactorial experimental designs and therefore insights into how the interaction of various environmental factors (e.g. temperature, food, medium) and anthropogenic stressors (e.g. microplastics, metals, pesticides) affects growth. New understanding, theory and tools for environmental risk assessment and management will follow.

## Methods

### Study design overview

Two types of experiments were conducted: one-time scanning of individual organisms of varying sizes and periodic scanning of a growing cohort. Organisms were imaged with the spheroid counter Cell^3^iMager (cc-5000, SCREEN Holdings Co. Ltd., Kyoto, Japan), a bright-field well plate scanner with automatic scanning, focus in z-dimension and image processing. One-time scanning experiments involved scanning individual organisms with the spheroid counter to measure a range of size related parameters and subsequently measuring individual organism size manually on a microscope. This enables construction of size calibration curves which will allow converting machine (Cell^3^iMager) measured size into conventional length measurements. Periodic scanning experiments involved maintaining individual organisms in well plates and repeatedly scanning the same individuals over time to measure their growth.

### Culturing of organisms for measurement

Organisms used for creation of size calibration curves were maintained at 18 degrees Celsius in low light conditions. Populations of *C*. *dubia* and *D*. *magna* species were maintained in 800 ml beakers filled with aerated Buxton still water (Nestlé UK Ltd, Rickmansworth, UK). Each beaker was supplied with one Daphnia food pellet per week (DTS125, Blades Biological Ltd, Cowden, Edenbridge, UK; for composition see Supporting Information). *H*. *azteca* stock cultures were maintained in a 60 L tank with limestone gravel sediment, in filtered and aerated tap water and fed with cucumber and fish food flakes (Bradshaws Pond Flakes, Bradshaws Ltd., York, UK). *A*. *aquaticus*, *Chironumus spec*. larvae and *Culex spec*. larvae were purchased from a supplier (DTS125, Blades Biological Ltd, Cowden, Edenbridge, UK) and stored in the water they were shipped with until measurement. No culturing of these organisms was carried out.

### Experimental conditions

Only *C*. *dubia* and *D*. *magna* were used in periodic scan experiments. Individual organisms, purchased as ephippia (resting eggs) from Microbio Tests Inc. (Mariakerke, Ghent, Belgium), were washed and placed in 200 ml aerated Buxton still water in beakers at 25 degrees Celsius and 6000 lux light intensity. Resting eggs were left to hatch for 80 hours, and individual daphnids were collected with a plastic mini pipette and transferred into well plates. The first eggs hatched after 72 hours, therefore, all daphnids were no older than 8 hours.

The daphnids were deposited into a 24 well microtiter plate (#83.3922, Sarstedt AG & Co, Nürnbrecht, Germany), with one individual per well. Medium transferred with the individual was removed and 2 ml of test medium was added. Test medium was made by adding 220 mg of daphnia food pellet to 1 L of either Buxton still water or Environmental Protection Agency Standard Freshwater^27^. The solution was mixed using a kitchen blender (1.5 L Cookworks Liquidiser) at full power for two minutes to break down the pellets and sieved subsequently to remove any particles larger than 125 μm. Plates were stored in a Sanyo Versatile Environmental Test Chamber at 20 degrees Celsius with a 16:8 hour light:dark cycle and placed into the spheroid counter daily for 2 hours of scanning.

Study medium was changed twice weekly to keep food levels constant and remove any offspring. Medium changes were carried out by filling up a new well plate with medium, then moving the largest individual in each well from the old plate into the newly filled plate. Organisms were transferred with a plastic micro pipette and moved with minimal medium from the old well.

### Spheroid counter (Cell^3^iMager) operation

As the vertical position of the organism in the well (i.e. the depth where the organism is in the well) varied between wells, a single scan was not guaranteed to obtain an in-focus image. Therefore, bracket scans, which scan the wells at multiple heights, were used to take images focusing at different levels to increase the chance of retrieving an in-focus image (Fig. 4). Scan settings (Table 2) for the scanning software (CellScan, Version 2.3.3.28, SCREEN Holdings Co. Ltd., Kyoto, Japan) were optimised during a preliminary experiment to determine the best software settings to scan each species. All images were scanned with a resolution of 2400 dpi, using a linear tone curve and with a bottom scanner height of 0 mm.

**Table 2:** Spheroid counter (Cell^3^iMager) scan settings for each study species.

Species	Well plate size (number of wells)	Top scanner height (mm)	Scan increments (mm)
*Daphnia species*	24	6.4	0.4
*Asellus aquaticus*	6	6.5	0.5
*Hyalella azteca*	24	6.5	0.5
*Chironomus spec*. *larvae*	24	6.4	0.4
*Culex spec*. *larvae*	24	6.4	0.4

Top scanner height shows the maximum height of scanned and scan increment is the distance the scanner moves vertically between scans.

For the larger species, *H*. *azteca* and *A*. *aquaticus*, we increased scanning intervals as fewer images at different heights were necessary to achieve an in focus image. Increasing image quality to 4800 dpi did not lead to significant improvement in measurement accuracy, but did increase both file size and time taken for scanning. Therefore, 2400dpi was used for all scans. *A*. *aquaticus* individuals were too large to be successfully scanned in a 24 well plate, thus were instead scanned in a 6 well plate.

Following a scan, the accompanying software (CellMeasureManager, Version 2.3.3.28, SCREEN Holdings Co. Ltd., Kyoto, Japan) automatically selected the highest quality image for approximately half of the scans. For scans where the automatic image selection via software was not successful, the user selected the best quality image. The software then analysed each well by applying a user defined image processing protocol (termed recipe by the CellMeasureManager software) to identify and measure objects. This recipe defined search area, object detection and object classification parameters to identify objects as living or debris.

### Microscope operation

For the creation of a calibration curve, individuals were measured immediately after scans. Individuals were removed from the well plates via plastic micro pipette (a small sieve was used for *A*. *aquaticus*) and placed on a glass slide. Excess water was then removed from the slide, using a 1 millilitre pipette, to fix the individual in place for measuring. *A*. *aquaticus* were too large to be transferred and were instead kept in the well plates with all the water removed. A Zeiss Axio Zoom. V16 (Jena, Germany) microscope was used to view organisms and capture an image for measurement of length. *H*. *azteca*, *D*. *magna* and *D*. *pulex* were viewed at ×26 magnification, and ×50 magnification was used for *C*. *dubia*. *Culex* spec. larvae, *Chironomus* spec. larvae and *A*. *aquaticus* were viewed at ×11.2 magnification.

**Figure 5:**
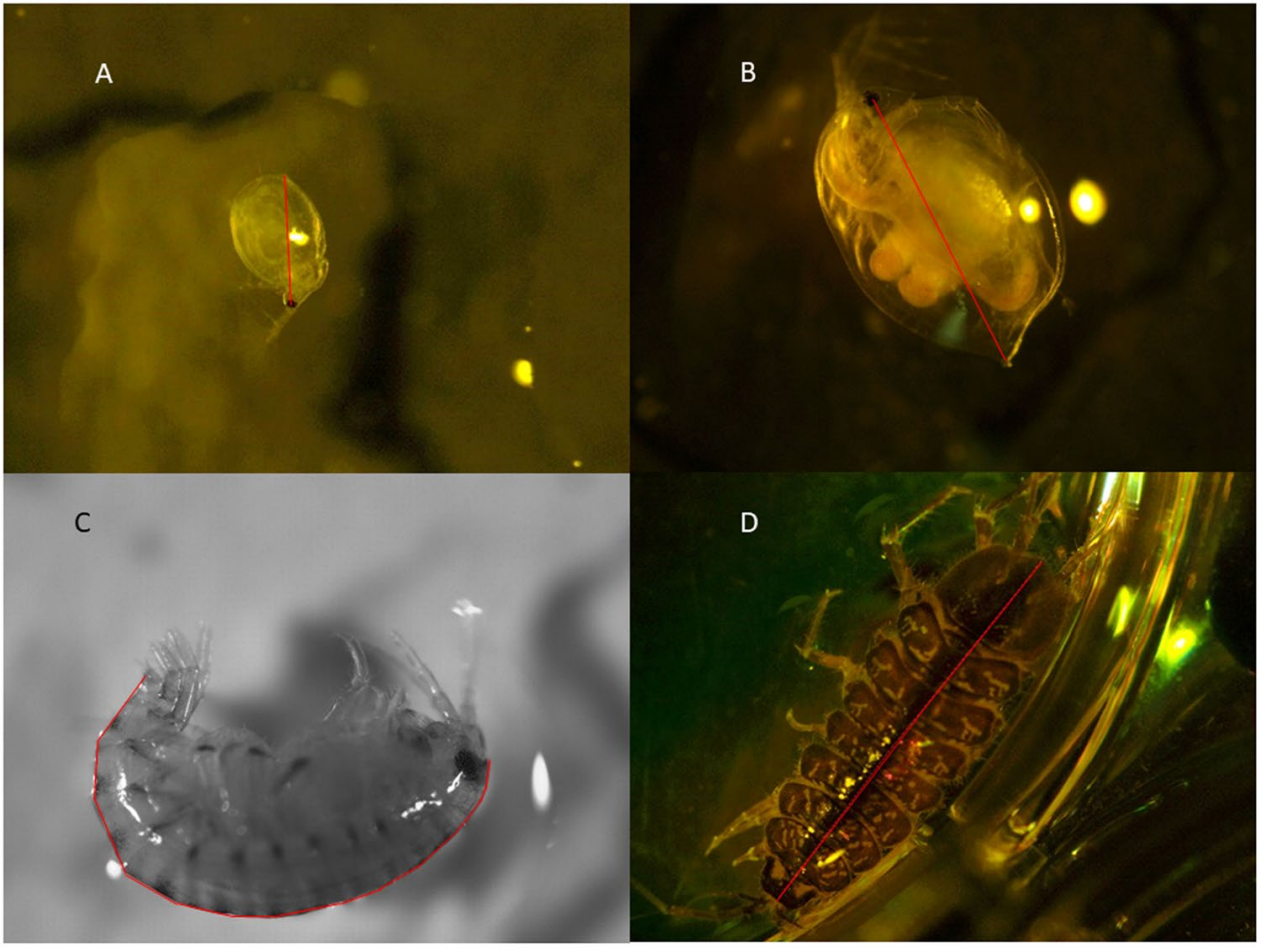
Length measurements for different species under the microscope. (**A**) *Ceriodaphnia dubia*, (**B**) *Daphnia magna*, (**C**) *Hyalella azteca*, (**D**) *Asellus aquaticus*. Red lines illustrate how the length of the organism was measured.

Length measurements were carried out using the Zeiss companion software to measure straight line distances. Both daphnid species’ length was determined by measuring from the centre of the eye to the base of the tail in a single line^15,28^ (Fig. 5A,B). Lengths of *H*. *azteca* were determined by following the dorsal length of an individual from the base of the first antenna to the tip of the third uropod^29^ (Fig. 1C). Approximately 15 lines were drawn along the dorsal length to follow the curve of each organism. The lengths of these lines were then summed to give *H*. *azteca* body length. *A*. *aquaticus* body length was measured from the top of the head to the tip of the Pleotelson^30,31^. *Culex* spec. larvae were measured from the top of the thorax to the tip of abdomen segment VIII following the curve of the organism. *Chironomus* spec. larvae total body length was defined as the measure from the top of the head to the tip of the tail, also following the curve of the organism.

### Data analysis

Data analysis was carried out using R^32^ version 3.5.0 with packages ggplot2^33^, GridExtra, ggpubr and rstudioapi used for the production of graphs. Regression analysis was used to determine the linear relationship between measurements from the microscope and spheroid counter. Strength of the regression equation was determined using an *r*^2^ value. Fits of the von Bertalanffy growth curves on individuals (Fig. 3) were performed in Matlab using the BYOM platform (http://www.debtox.info/byom.html).

## Supplementary information

Supporting information

Dataset 1

Dataset 2

## Data Availability

All data from this study is available in the supplementary information files.
