# Supplementary material for: Automated, high-throughput measurement of size and growth curves of small organisms in well plates
Source: Sci Rep. 2019 Jan 9;9:10. doi: 10.1038/s41598-018-36877-0 (PMC6327043; doi:10.1038/s41598-018-36877-0)
Supplement: Supplementary file 1 — Supporting information [file 41598_2018_36877_MOESM1_ESM.pdf]

Supporting Information to

Automated, high-throughput measurement of size and growth curves of small organisms in well plates

James Duckworth[1], Tjalling Jager[2] & Roman Ashauer[1]*

1) Environment Department, University of York, Wentworth Way, Heslington, York, YO10 5NG, United Kingdom

2) DEBtox research, De Bilt, The Netherlands

*) Corresponding author: roman.ashauer@york.ac.uk

Composition of daphnia food pellets

Analytical Constituent:

Crude Oils & Fats 21%
Crude Protein 37.5%
Crude Ash 5.6%
Crude Fibre 4.5%
Calcium 0.7%
Phosphorus 0.8%
Sodium 0.2%

Composition:

Wheatfeed, Soya (bean) protein concentrate, Fish meal, Rape seed oil, Vital Wheat gluten, Fish oil, Sunflower seed expeller (organically grown)
Horse beans dehulled, Vitamins, Minerals, Mono ammonium phosphate, Methionine

Additives (per kilo):
Trace elements

E1 Iron (Ferrous Sulphate monohydrate) 40mg
E2 Iodine (Calcium iodate anhydrous) 2mg
E4 Copper (Cupric sulphate pentahydrate) 5mg
E5 Manganese (Manganese sulphate monohydrate) 15mg
E6 Zinc (Zinc sulphate monohydrate) 100mg
Antioxidants
E3 10 Propyl gallate
E3 20 BHA (Butylated hydroxyanisole) 2mg
E3 21 BHT (Butylated hydroxytoluene) 74mg
Vitamins
E6 71 Vitamin D3 1500 IU
E6 Vitamin A 4000 IU
